# Case Report: Management of a Patient With Chylomicronemia Syndrome During Pregnancy With Medical Nutrition Therapy

**DOI:** 10.3389/fnut.2021.602938

**Published:** 2021-03-05

**Authors:** Maryam Zahedi, Golaleh Asghari, Parvin Mirmiran, Farhad Hosseinpanah

**Affiliations:** ^1^Obesity Research Center, Research Institute for Endocrine Sciences, Shahid Beheshti University of Medical Sciences, Tehran, Iran; ^2^Nutrition and Endocrine Research Center, Research Institute for Endocrine Sciences, Shahid Beheshti University of Medical Sciences, Tehran, Iran

**Keywords:** medical nutrition therapy, hypertriglyceridemia, pregnant women, GDM, chylomicronemia syndrome

## Abstract

**Background:** Hypertriglyceridemia (HTG) during pregnancy may be accompanied by acute pancreatitis, hyperviscosity syndrome, and preeclampsia. HTG during pregnancy should be managed by a multidisciplinary team; however, no clinical guidelines exist for severe gestational HTG.

**Case Presentation:** We herein present a case of a 36-year-old in the first pregnancy (G1P0Ab0), with a history of severe HTG-induced necrotizing pancreatitis 9 years earlier. There was no family history of HTG. During these years, she did not follow any appropriate diet or medical therapy for HTG. She became pregnant in May 2019, without preconception counseling. Eruptive and tuberoeruptive xanthomas appeared in the 27th week of pregnancy. Serum triglycerides (TGs) and fasting blood sugar (FBS) were 6,620 and 124 mg/dL, respectively, indicating HTG and gestational diabetes (GDM). After admission for the management of severe HTG, she was put on parenteral nutrition with dextrose water 5% and infusion insulin therapy without receiving any enteral carbohydrate for 2 days. Following that, a very low-fat diet and omega-3 fatty acids (1,200 mg/day) were started. After 4 weeks, TG levels reached 1,000 mg/dL, and her self-monitoring blood glucose levels showed appropriate blood glucose for pregnancy. She underwent a successful elective cesarean section in the 39th of pregnancy.

**Conclusion:** This case report demonstrates that HTG during pregnancy could be managed by medical nutrition therapy (MNT).

## Background

Hypertriglyceridemia (HTG) can arise from hepatic overproduction, decreased clearance of chylomicrons, and very-low-density lipoprotein (VLDL) remnant, ineffective lipolysis, or a combination of these factors (1). Severe HTG is defined as TG concentration >885 mg/dL, which is almost accompanied by chylomicronemia's pathological presence (2). Chylomicronemia manifested by eruptive xanthomas, lipemia retinalis, hepatosplenomegaly, and episodes of pancreatitis called chylomicronemia syndrome (3). Severe HTG is most often due to polygenic susceptibility interacting with secondary non-genetic factors (4), including consumption of high-fat foods, alcohol, estrogen-containing medication, pregnancy, obesity, diabetes, or medications that increase VLDL secretion (e.g., steroids and beta-blockers) (4, 5).

During pregnancy, significant alterations to lipid homeostasis occur (4) include an increase in TG and total cholesterol levels, which are mediated by estrogen, progesterone, and human placental lactogen (HPL) (6). The presence of HTG in pregnancy may cause other health-threatening risks, including acute pancreatitis, hyperviscosity syndrome, and preeclampsia (1).

There are no strict clinical guidelines for the treatment of HTG during pregnancy. Generally, during the prenatal period, a low-fat and low-glycemic diet with adequate nutrients to avoid essential fatty acid deficiency is advised. In refractory cases, hospitalization for parenteral nutrition or intravenous insulin therapy, fibrate use after the first trimester, and plasmapheresis are considered (1, 6). Unfortunately, only a few studies address the management of HTG during pregnancy through medical nutrition therapy (MNT).

## Case Presentation

**Figure 1:**
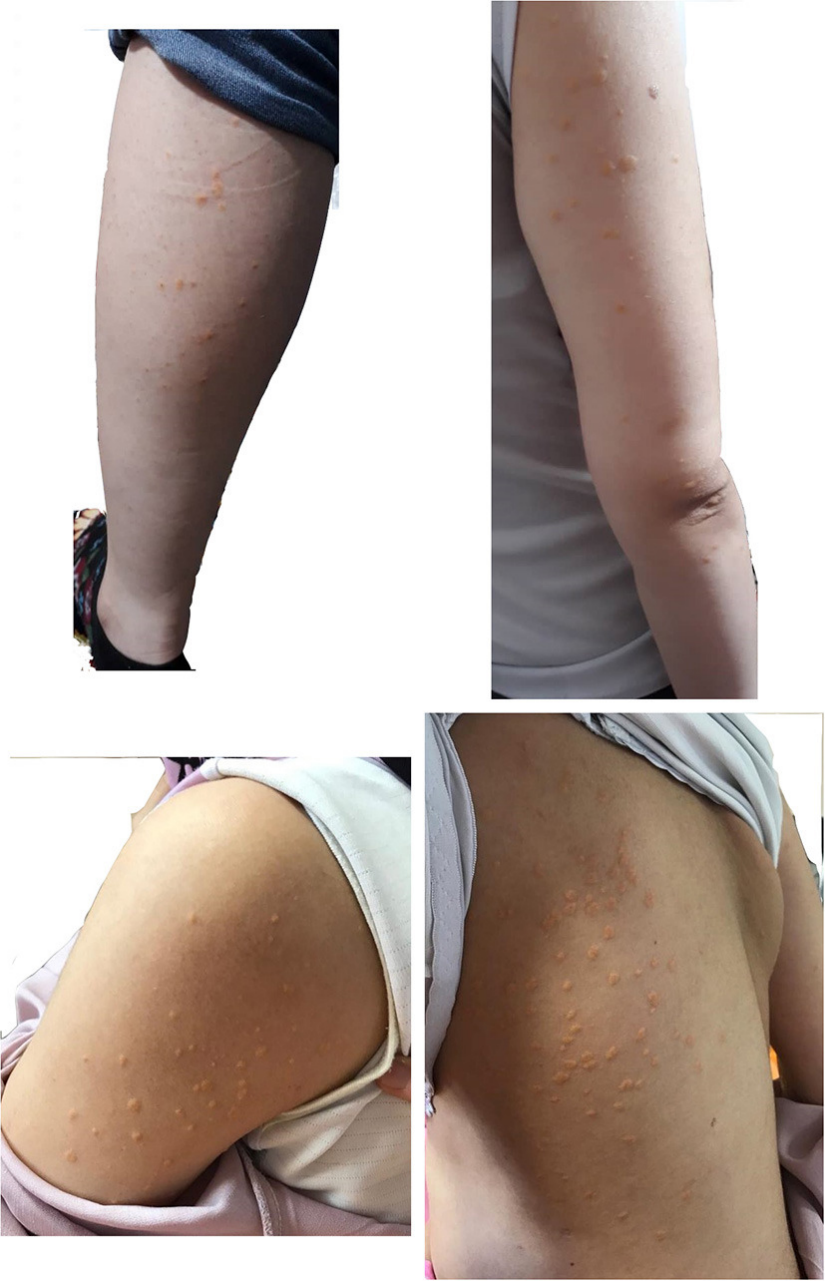
Eruptive and tuberoeruptive xanthomas.

We herein present a case of a 36-year-old in the first pregnancy (G1P0Ab0), with a severe HTG presented in the second trimester of pregnancy. Eruptive xanthomas in the forearms, legs, back, and tuberoeruptive xanthomas on her elbows (Figure 1) occurred in the 27th week of pregnancy, and she was admitted to a hospital in November 2019. Her serum lipoprotein profile was as follows: fasting TG: 6,620 mg/dL, total cholesterol: 459 mg/dL, high-density lipoprotein cholesterol (HDL-C): 76 mg/dL, and serum amylase and lipase levels were within normal limits. Except for pregnancy, no other aggravating factors for HTG were found. After admission, TG level reached 8,683 mg/dL. Fasting blood sugar (FBS) was normal in early pregnancy. However, the glucose tolerance test (GTT) with 75 g anhydrase glucose showed: FBS: 124 mg/dL, 1-h glucose: 168 mg/dL, 2-h glucose: 130 mg/dL indicating the presence of gestational diabetes. There were no thyroid, liver, and renal function abnormalities. Abdominal ultrasound imaging was unremarkable with no evidence of pancreatitis and hepatosplenomegaly.

She had a hospital admission history 9 years ago with acute abdominal pain (acute necrotizing pancreatitis) and severe HTG >6,000 mg/dL. She had been discharged without symptoms. During her irregular medical visits, occasional fibrates were prescribed by her physician. She did not report severe HTG in her first and second-degree family. At the beginning of pregnancy, her serum TG level was 619 mg/dl with normal fasting blood glucose (FBG). Pre-pregnancy weight and body mass index (BMI) were 52 kg and 19.5 kg/m^2^, respectively. She still did not have a specific diet and did not consult an endocrinologist or dietitian for HTG because she had a low socioeconomic status. Considering the history of necrotizing pancreatitis as well as a lack of family history of HTG and access to genetic testing, our differential diagnosis based on clinical evidence was rare monogenic HTG.

**Figure 2:**
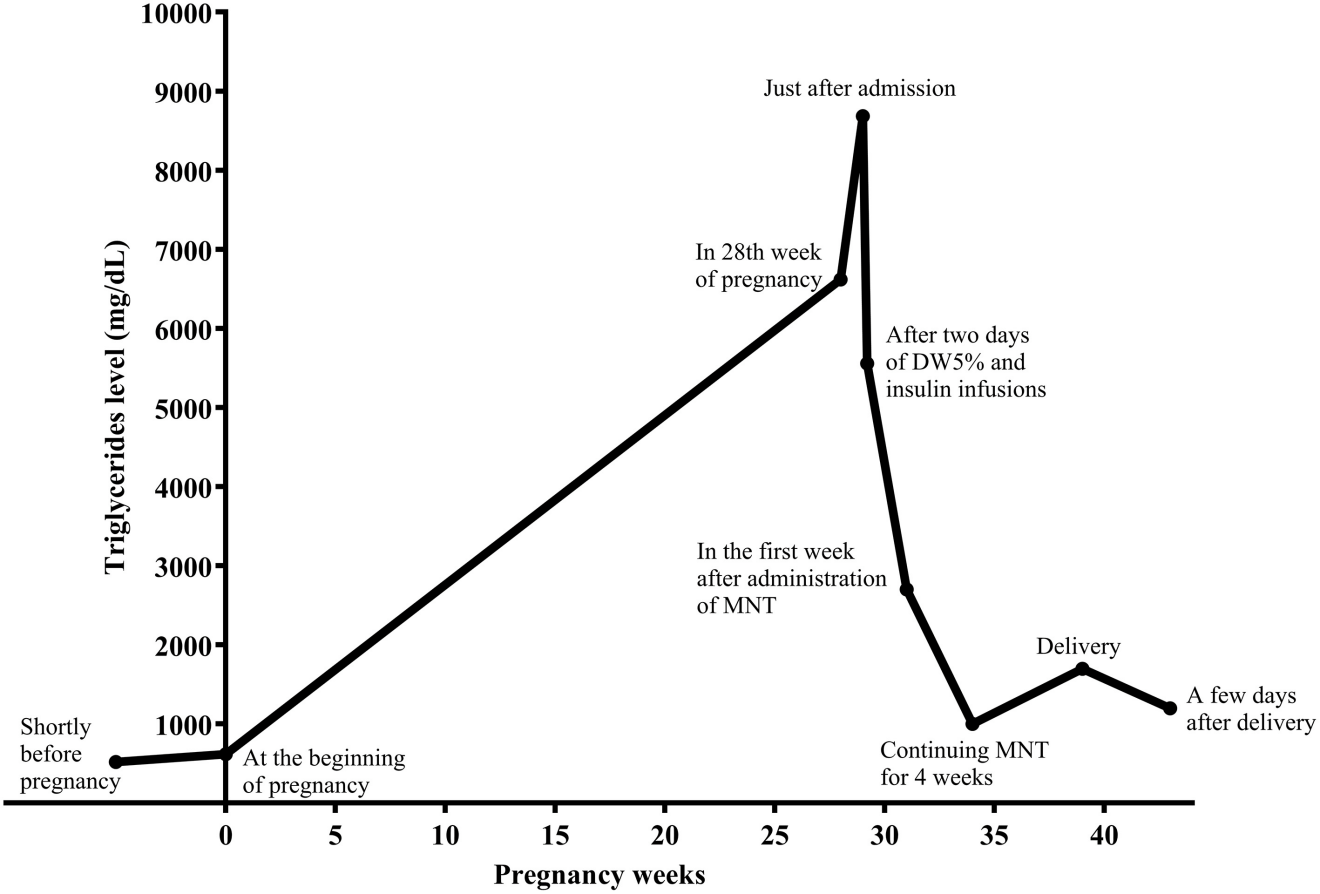
Serum triglyceride (TGs) concentration during pregnancy and postpartum.

To reduce TGs rapidly, the patient was put on dextrose 5% in water (D5W, 2,500 mL per day) and insulin regular (5–7 unit/hour) infusion, without any enteral intake, and with close monitoring for blood glucose and serum electrolytes, including sodium and potassium levels. After 2 days, TG level was 5,560 mg/dL. Insulin infusion was discontinued. A low-fat diet consisted of 10, 25, and 65% of energy from fat, protein, and carbohydrate, respectively, was prescribed by the dietician. The diet comprised four servings of skim-fat milk or yogurt, four servings of vegetables, six servings of fruits, one serving of simple carbohydrates such as sugar or honey, and 11 servings of grains. Three servings of meat and its substitutes were prescribed, including plant-based proteins such as legumes, one serving from egg white, and other servings from white meat, including chicken and turkey. Concurrently, seven capsules daily of omega-3 fatty acids containing 360 mg eicosapentaenoic acid (EPA) and 240 mg docosahexaenoic acid (DHA) per capsule (equal to 4,200 mg) were started in divided doses. Nevertheless, due to cost and availability, the patient received omega-3 at a dose of five capsules daily (equal to 3,000 mg) for 10 days and then two capsules daily (equal to 1,200 mg) for the rest of the pregnancy. She was followed by a multidisciplinary team consisting of an endocrinologist, obstetrician, and a dietitian. In the 1st week after MNT administration and omega-3 fatty acids, TG levels dropped to 2,700 mg/dL (Figure 2).

Self-monitoring of blood glucose revealed a mean level of FBS and 2-h plasma glucose equal to 74 mg/dL and 96 mg/dL, respectively. She was discharged with a 10% fat-restricted diet with medium-chain TGs (MCT) 15 g daily. Continuing MNT for 4 weeks, TG levels reached 1,000 mg/dL, and all SMBGs were in the recommended ranges for the pregnancy. From the time of admission to the specialists and up to labor, she had regular weekly contacts with her endocrinologist and dietician. She underwent elective cesarean section at 39th week of pregnancy with TG level of 1,700 mg/dL. She delivered a healthy baby girl weighing 2,750 g. At the end of her pregnancy, the body weight was 58 kg (equal to 6 kg weight gain). The patient had an uncomplicated postoperative course and was discharged and recommended to continue her diet. A few days after delivery, her TG level dropped to 1,200 mg/dL. She was advised to breastfeed her baby. No complications or health problems related to HTG was found up to 2nd month of postpartum.

## Discussion

Our patient was a 36-year-old pregnant woman presenting chylomicronemia syndrome during pregnancy, which seemed to be managed successfully by MNT. The widely used term familial chylomicronemia syndrome is synonymous with monogenic chylomicronemia (7). The absence of secondary factors and HTG diagnosis at young adulthood suggests monogenic chylomicronemia, mainly if hypertriglyceridemia is severe and associated with pancreatitis (8).

Guidelines advise a combination of restricting dietary fat intake with drug treatment (e.g., fibrates, niacin, or omega-3 fatty acids) to manage severe HTG in non-pregnant patients. However, no clear clinical guidelines exist for severe gestational HTG to maintain the balance between maternal and fetal needs. We suggest that a multidisciplinary team should manage women with gestational HTG (8, 9), as it is associated with many clinical challenges (4).

A few case reports are expressing MNT to manage severe HTG among pregnant women. Shenhav et al. treated a pregnant woman admitted at 33rd weeks of pregnancy with TG concentration of 13,805 mg/dL with a diet consisting of intravenous hydration with 3,000 mL of 5% dextrose and a hypocaloric low-carbohydrate, low-fat diet. The serum concentration of TG was reduced to 1,035 mg/dL after 27 days of the intervention (10). Furthermore, Kleess et al. reported a case of a female aged 38-year in the 21st week of pregnancy with GDM and severe HTG (TGs and FPG 7,812 and 300 mg/dL, respectively). She had received insulin glargine and lispro and a diet consisting of low-fat (<20% of calories) and low-carbohydrate. At the delivery, the serum concentration of TGs was <2,000 mg/dL (11). None of the previous studies indicated the MNT in detail, as we have provided in the current study.

Many factors and presenting symptoms are involved in the admission of patients with HTG (6). The patient was admitted due to rapid TG increase, poor dietary compliance, previous pancreatitis due to HTG despite the absence of present pancreatitis and presence of GDM (6). Short-term hospitalizations can be used proactively to reduce TG levels through parenteral nutrition (PN) (10, 12), or intravenous insulin therapy (13) if GTT is also impaired, injection of heparin (14, 15), and plasmapheresis (16–18) (Table 1). The literature showed that MNT's effect on severe HTG is acceptable; however, its effect would not be acute, and serum concentration of TG cannot rapidly decrease.

**Table 1:** Literature review in case reports preenting a therapy in a pregnant female with severe hypertriglyceridemia (HTG).

**Author and year**	**Admission time to hospital**	**Intervention**	**Results**	**Conclusions**
Kleess el al., 2018 (11)	21st weeks of pregnancy	Insulin along with low-fat (<20% of calories) and low-carbohydrate diet	TG reduced from 7,812 to 1,800 mg/dL	A multidisciplinary team, along with a dietitian, can effectively improve severe HTG.
Safi et al., 2014 (16)	28th weeks of pregnancy	Plasmapheresis and intravenous heparin	TG reduced from 2,661 to 608 mg/dL after 5 days	Plasmapheresis for rapid reduction of TG and heparin for keep TG low
Gupta et al., 2014 (17)	38th weeks of pregnancy	Plasmapheresis and fibrate derivatives as well as low fat, restricted calorie clear liquid diet.	TG reduced from 12570 to 295 mg/dL after 8 days	Plasmapheresis is an intensive treatment to reduce TG in patients with HTG dramatically
Basar et al., 2013 (18)	21st weeks of pregnancy	Medical nutrition therapy, ω-3 fatty acids 3 g/day, and NPH insulin were started and continued with plasmapheresis	TG reduced from 12,000 mg/dL to 1,121 mg/dL after 12 days	Plasmapheresis was more effective than medical nutrition therapy and insulin infusion
Vandenbroucke et al., 2008 (15)	37th weeks of pregnancy	A course of heparin and a low-fat diet	TG reduced from 84,470 to 240 mg/dL after 6 days	Heparin and a low-fat diet can quickly decrease triglyceridemia and the healing of acute pancreatitis.
Gürsoy et al., 2005 (13)	37th weeks of pregnancy	Continuous intravenous insulin–glucose	TG reduced from 10,092 to 608 mg/dL after 5 days	Intravenous insulin and cessation of oral intake leads to dramatically decreased the triglyceride levels
Sleth et al., 2004 (14)	37th weeks of pregnancy	A single dose of heparin per day and a very low-fat diet	TG reduced from 950 to 100 mg/dL after 48 h	Heparin along with intravenous nutrition is immediate action in the management of severe HTG
Shenhav et al., 2002 (10)	33rd weeks of pregnancy	Intravenous hydration with 3,000 mL of 5% dextrose and a hypocaloric low-carbohydrate, low-fat diet containing 10 g fat per day and MCT	TG reduced from 13,805 to 1,035 mg/dL after 27 days	Dietary intervention was helpful; however, its effect not acute.
Sanderson et al., 1991 (12)	33rd weeks of pregnancy	Intravenous fluids with the reinstitution of a low-fat diet.	TG reduced from 6,637 to 265 mg/dL after 7 days	Parenteral nutrition with a low-fat content was enough to improve HTG

Starting PN is effective because lipids' systemic delivery bypasses the portal system, allowing for peripheral metabolism and trans-placental passage of fats (1). Moreover, insulin is a rapid and potent activator of LPL. It can be used to treat severe HTG because it increases the removal of TGs from the plasma. Given the risk of hypoglycemia, it is usually co-administrated with glucose infusion (4). Therefore, after the patient was hospitalized, we prescribed D5W and regular insulin infusion through two separate veins. Nevertheless, no significant drop in TG levels was observed after 2 days (Figure 2).

In non-pregnant individuals, higher doses of omega-3 fatty acids that contain EPA and DHA (4 g/day total EPA+DHA) have been shown to reduce ongoing metabolic control, the concentration of VLDL, and possible chylomicron secretion (2, 19). Omega-3 fatty acids are the cornerstone of safe therapy for mother and child in the long-term (4). As previously mentioned, due to cost and limited availability, our patient received only 1,200 mg of omega-3 daily. Therefore, the triglyceride drop does not appear to be related to omega-3.

Dietary intervention with fat <20% (4), ideally, <10% of calories should be immediately started (5). However, adherence to such a regimen is extremely challenging for most patients (5). In addition, restricted fat intake can lead to maternal and, more importantly, fetal deficiency of omega-3 and omega-6 essential fatty acids (EFA) (including alpha-linolenic acid (ALA) and linoleic acid (LA)) as well as the vital long-chain omega-3 PFA (including EPA and DHA). Although symptoms of EFA deficiency are relatively mild for the mother (typically skin dryness and desquamation) (4), fetal EFA and long-chain polyunsaturated fatty acids deficiency may end in impaired fetal brain and visual development (4). The diet should include a minimum of 300 mg of EPA and DHA (4). The use of omega-3- fatty acids and medium-chain triglycerides (MCTs) will provide calories while preventing plasma TGs increase (4, 5). Although MCTs' safety has not been specifically evaluated in the fetus (20), in a randomized, double-blind intervention by Rayyan et al. MCT- containing emulsion was safe and well-tolerated by preterm infants (20). The patient was discharged with a low-fat diet, containing mostly medium-chain triglyceride foods. In addition to attention to the fat in a diet, avoidance of refined carbohydrates, especially simple sugar, should be a part of a dietary intervention (18). Compelling evidence indicated that foods contain simple sugars and fructose supply substrates for TG production, which substantially are enhancing TG levels in susceptible people (21, 22). Therefore, dietary intake of the patients was limited to only one serving per day simple sugar and the rest of carbohydrate was provided by whole-grain foods. We strongly encouraged her to refrain from eating any food with added sugar by educating food labeling.

Although metabolic control is ongoing, TG concentrations may rapidly rebound at any time (2). The risk of pancreatitis is always present, and more effective therapies are required (2). Close surveillance of plasma TG concentrations during pregnancy is essential and should be followed up at least on a monthly basis. As pregnancy progresses, TG levels may need to be monitored every 1–2 weeks (4); we carried this out with our patient. Strong consideration should be given toward induction once fetal maturity is established (i.e., at 36th weeks), especially in women whose TG levels show a steep upward trend in the third trimester (4). However, as previously discussed, due to the low fetal growth for her gestational age, she underwent an elective cesarean section with meticulous fetal and maternal monitoring at the 39th week of pregnancy.

In the postpartum period, the decision to resume these medications will depend on the latest blood results and trend of the serum TGs as well as maternal breastfeeding plans (4). In the postpartum period, considering the relative reduction of TGs, our patient continued to receive individualized MNT.

## Conclusion

In the current study, the case with severe gestational HTG, who was at high risk for pancreatitis, seemed to be treated with MNT successfully. The management led to the termination of a safe pregnancy and the birth of a healthy baby girl. Hopefully, we will see more case studies and in the future, so we can begin to provide recommendations on MNTs that could be acceptable for pregnant women.

## Data Availability Statement

The original contributions generated for this study are included in the article/supplementary material, further inquiries can be directed to the corresponding author/s.

## Ethics Statement

The patient provided her written informed consent to participate in this study. Written informed consent was obtained from the patient for the publication of any potentially identifiable images or data included in this article.

## Author Contributions

MZ and FH interpreted in the diagnosis of disease, medical treatment, and follow-up of the patient. GA and PM contributed to MNT. All authors read and approved the final manuscript.

## Conflict of Interest

The authors declare that the research was conducted in the absence of any commercial or financial relationships that could be construed as a potential conflict of interest.

## Abbreviations

HTG: Hypertriglyceridemia
TGs: Serum triglycerides
FBS: Fasting blood sugar
GDM: Gestational diabetes
MNT: Medical nutrition therapy
VLDL: Very low-density lipoprotein
WHO ICD: World Health Organization International Classification of Diseases
HPL: Human placental lactogen
BMI: Body mass index
HDL-C: High-density lipoprotein cholesterol
GTT: Glucose tolerance test
D5W: Dextrose 5% in water
EPA: Eicosapentaenoic acid
DHA: Docosahexaenoic acid
EFA: Essential fatty acids
ALA: Alpha-linolenic acid
LA: linoleic acid
MCTs: Medium-chain triglycerides.
